# Impact of global warming scenarios on life-history traits of *Tetranychus evansi* (Acari: Tetranychidae)

**DOI:** 10.1186/s12898-019-0264-6

**Published:** 2019-11-27

**Authors:** Noureldin Abuelfadl Ghazy, Tetsuo Gotoh, Takeshi Suzuki

**Affiliations:** 1 Graduate School of Bio-Applications and Systems Engineering, Tokyo University of Agriculture and Technology, Koganei, Tokyo 184-8588 Japan; 2 Department of Agricultural Zoology, Faculty of Agriculture, Mansoura University, El-Mansoura, 35516 Egypt; 3 Faculty of Economics, Ryutsu Keizai University, Ryugasaki, Ibaraki 301-8555 Japan

**Keywords:** Biological invasion, Climate change, Life table, Pest, Simulation, Tomato red spider mite

## Abstract

**Background:**

The tomato red spider mite, *Tetranychus evansi* Baker & Pritchard (Acari: Tetranychidae), is an agricultural pest of solanaceous crops. Although *T. evansi* is of South American subtropical origin, it has recently expanded its distribution range to many tropical and temperate areas around the world. Its potential distribution range in response to scenarios of global warming was recently modeled, confirming its current and possible future distributions. Here, we experimentally investigated the biological traits of *T. evansi* in the context of the current and future global warming (2100) scenarios. Using an environmental simulation system, we tested the life-history traits of *T. evansi* under current summer temperatures (as of June, July, and August 2016) and under expected temperature increases based on two IPCC scenarios: RCP2.6 (+ 1 °C) and RCP8.5 (+ 3.7 °C). The mites were introduced into each scenario on 1 June and their sequential progeny were used for testing in each following month.

**Results:**

The mite could develop and reproduce under all scenarios. There was a decrease in the duration of lifespan and female fecundity at RCP8.5 during June and August, but this may be compensated for by the high intrinsic rate of increase, which implies faster population growth and shorter generation time.

**Conclusion:**

Our study and other reports reveal the high adaptability of *T. evansi* to a wide range of summer temperatures; this may explain its current distribution. We anticipate that global warming will favor the spread of *T. evansi* and may further expand its distribution to a large area of the globe. These findings should be of ecological and practical relevance for designing prevention and control strategies.

## Background

Global warming is expected to have measurable impacts on terrestrial organisms [1–4]. According to the Intergovernmental Panel on Climate Change (IPCC) and Representative Concentration Pathways (RCPs) for greenhouse gas concentration scenarios, the global mean surface temperature is projected to rise by 0.3 to 4.8 °C by the end of the 21st century [5]. Biological consequences of global warming may include invasion and extinction rate acceleration; interruption of biological timing (reproduction, migration, growing season length, outbreaks, and distributions); disruption of ecological interactions (phenology, food sources, and predators); and alteration of ecosystem composition and functions [1, 6–8]. However, the magnitude of these effects will depend largely on a species’ ability to adapt and cope with global warming or to migrate into more suitable environments [6, 9]. Therefore, understanding the way organisms may adjust to global warming is a key challenge to research into climate change and biological invasion. To visualize the impacts of, and species adjustments to, global warming, accumulation of detailed ecological and biological data are important for predicting potential problems and formulating appropriate countermeasures to conserve rare species and protect against invasive ones.

The tomato red spider mite, *Tetranychus evansi* Baker & Pritchard (Acari: Tetranychidae), was reported for the first time in Brazil by Silva [10]. At the time, the species was misidentified as *Tetranychus marianae* McGregor, but it was later described as *T. evansi* by Baker and Pritchard [11]. For a summary of the taxonomic status of *T. evansi*, see Navajas et al. [12]. *Tetranychus evansi* is a pest that is destructive to several economically important members of the family Solanaceae, including tomato, eggplant, potato, tobacco, and other nightshade species [13–15]. Recently, *T. evansi* emerged as an invasive pest distributed in the tropical and temperate areas of nearly 43 countries; it is associated with 136 host plants of 36 plant families [16–19]. Although *T. evansi* is not a serious pest in its native habitat, it causes severe losses—sometimes reaching 100%—in tomato and other solanaceous plants, and it also disrupts the community composition of other *Tetranychus* species in invaded areas [18, 20, 21]. *Tetranychus evansi* does not enter diapause and is able to produce throughout the year if environmental conditions are favorable [13, 14]. Characteristics such as a higher intrinsic rate of increase (particularly at high temperatures: the thermal optimum is ~ 35 °C) than other *Tetranychus* species, absence of effective biological control agents, resistance to pesticides, and the ability to manipulate plant defenses account for the high invasive potential of *T. evansi* [15, 20, 22–26].

In Japan, *T. evansi* was first reported in Osaka in 2001. Its distribution has now expanded throughout the country, particularly in areas with temperate or tropical climates [18, 23, 27]. According to Boubou et al. [28, 29], two distinct genetic lineages of *T. evansi* have colonized areas outside their region of origin: Lineage 1 has the most invasive potential and has been recorded in several countries around the world, whereas lineage 2 has been found only in some parts of southern Europe. Accordingly, the population of *T. evansi* in East Asia (including Japan) belongs to lineage 1. This lineage has broader adaptability to the climatic gradient in temperate ecosystems and can exploit a wider range of host plants than lineage 2. It might have been accidentally introduced into Japan from neighboring countries in Asia (e.g., Taiwan) or from African or European countries [12, 17, 18, 30–32].

Predicting the geographical distribution range of a species such as *T. evansi*, with its strong invasive abilities, is essential [18]. Using available data, Meynard et al. [31] statistically predicted the current distributions of *T. evansi* in its native range and its invaded range, as well as its expected future expansion range in response to climate change. However, to date, there has been no conclusive, solid, and empirical evidence demonstrating the likely impacts of global warming on the biological activities of *T. evansi*. Assessing population growth, survival, reproduction, and the rate of increase, as influenced by scenarios of global warming, on an individual species basis could be a valid approach [2]. Here, we experimentally examined the life-history traits of *T. evansi* under three temperature scenarios: the current temperature, plus two global warming scenarios according to the IPCC [5], namely the mean values for “a stringent mitigation scenario” (RCP2.6) and “high greenhouse gas emissions” (RCP8.5) in 2100. For the current temperature scenario, Tokyo summer (June, July, and August) temperatures of 2016 were used. For the future scenarios, current temperatures were increased by 1 °C to represent RCP2.6 or by 3.7 °C to represent RCP8.5. We selected summer because this is the season of greatest population expansion of several mite species in the field [33–37].

## Results

### Accuracy of temperature simulation

**Fig. 1:**
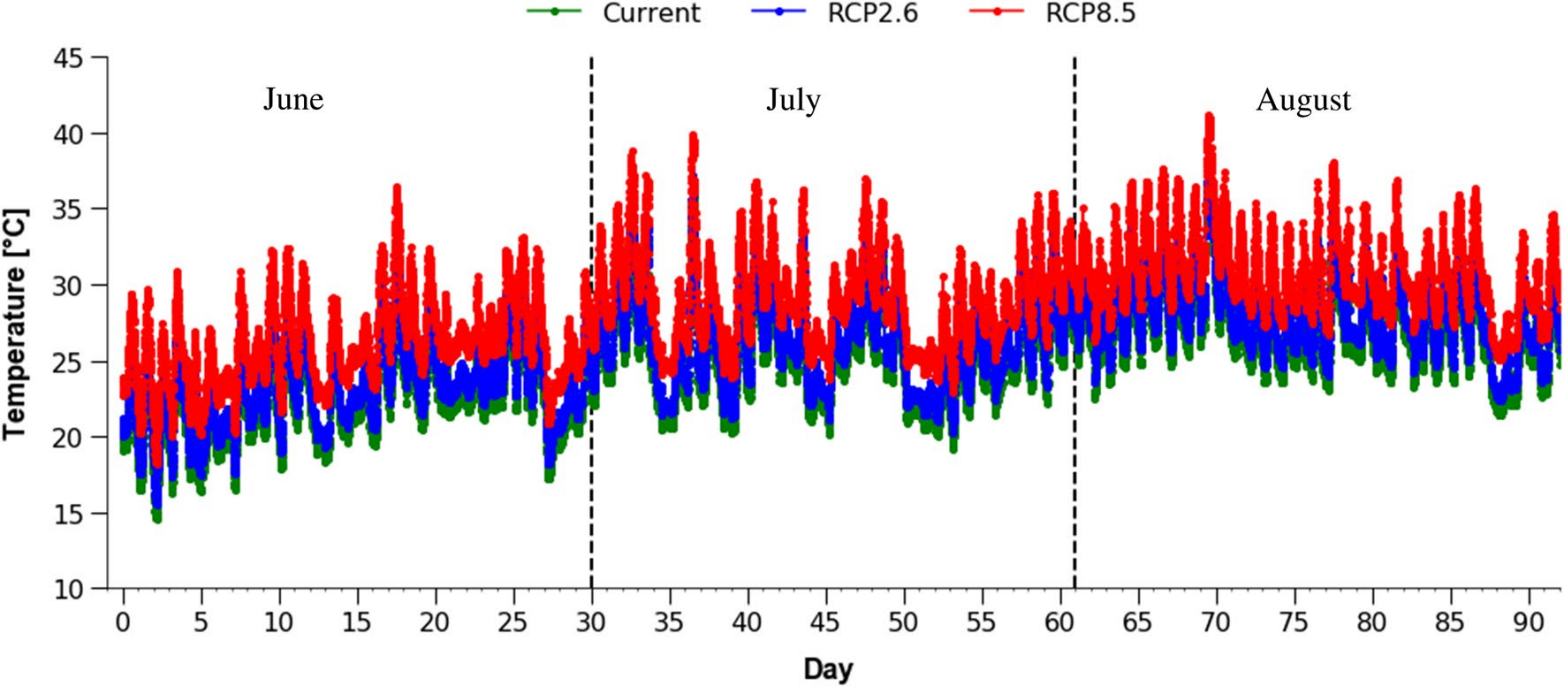
Simulated global warming scenarios. “Current,” natural temperature measured at 10-min interval (Japan Meteorological Agency); RCP2.6, current temperature increased by 1 °C; RCP8.5, current temperature increased by 3.7 °C. Mites were introduced to these conditions on 1 June and their sequential progeny were used in each subsequent month

**Fig. 2:**
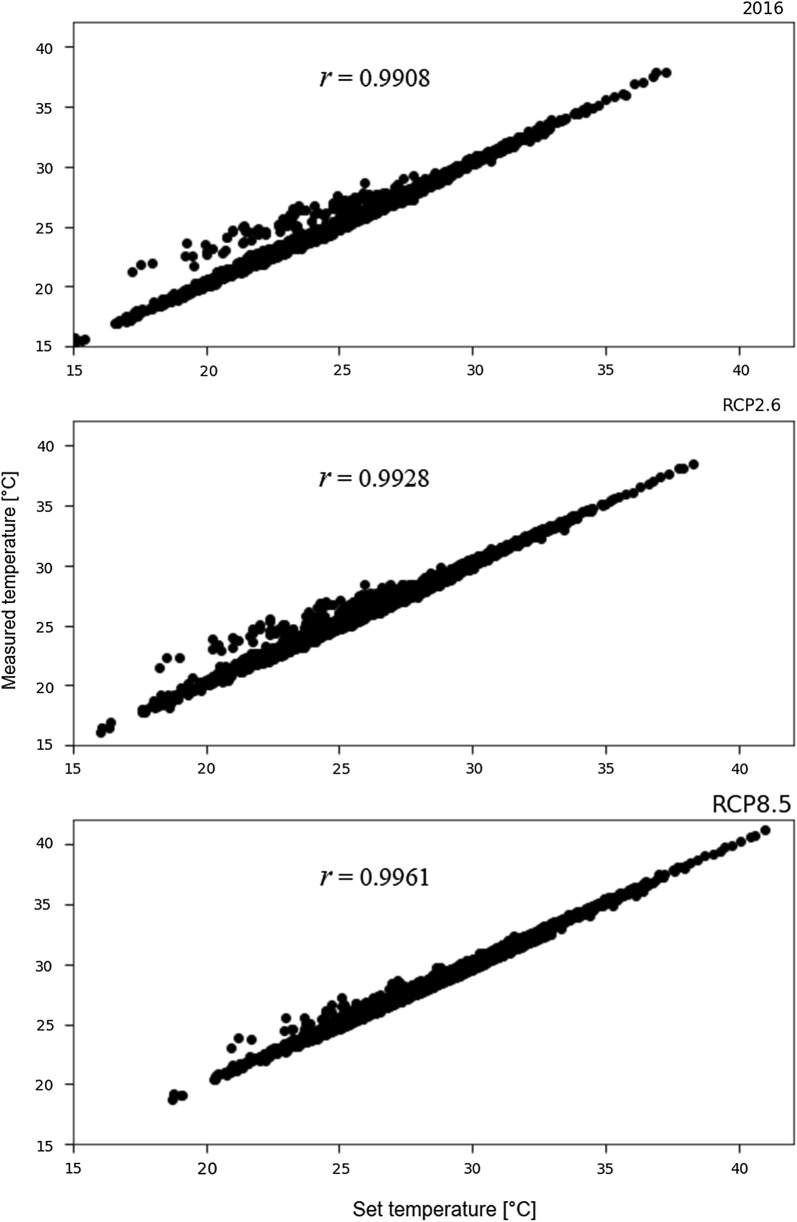
Pearson correlation coefficient (*r*) of the relationships between set and measured values of temperature in the simulation system under the three scenarios

The set temperatures values under the current and 2100 IPCC scenarios are shown in Fig. 1. More than 98% of the variance in temperature set values could be explained by changes in the measured values (Fig. 2).

### Relative humidity

Relative humidity was not controlled (Additional file 1: Figure S1). The measured relative humidity during the experiment was ~ 80%, ~ 70%, and ~ 65% under the current, RCP2.6, and RCP8.5 scenarios, respectively.

### Development and lifespan

Two-way ANOVA for the impact of month and RCP scenario on immature development time, lifespan, and reproduction (eggs/female) showed significant main effects and interactions of both factors (Table 1). Different temperature scenarios and months did not have significant effects on hatchability percentage (% hatch) or survival to adulthood (% survival) of immature *T. evansi* (χ^2^, *P* > 0.05, Table 2).

**Table 1:** Two-way ANOVA of factors (months and Representative Concentration Pathway [RCP] scenarios) affecting immature development time (egg-to-adult), lifespan, and reproduction of *Tetranychus evansi*

Parameter	Source	df	MS	*F*	*P*
Egg-to-adult	Month	2	34.74	3992.26	< 0.001
	RCP	2	8.03	922.94	< 0.001
	Interaction	4	0.19	21.33	< 0.001
	Residuals	484	0.01	–	–
Lifespan	Month	2	33.17	41.98	< 0.001
	RCP	2	29.65	37.53	< 0.001
	Interaction	4	3.86	4.89	0.001
	Residuals	469	0.79	–	–
Reproduction	Month	2	67.16	11.58	< 0.001
	RCP	2	131.42	22.66	< 0.001
	Interaction	4	81.21	14.00	< 0.001
	Residuals	422	5.80	–	–

**Table 2:** Effects of simulated global warming scenarios on number of days to development at each stage, survival rate to adult, and adult longevity and lifespan of *Tetranychus evansi*

Parameter	Global warming scenario					
	*n*	Current	*n*	RCP2.6	*n*	RCP8.5
June						
Hatchability (%)	129	96.9 ± 1.5 a	176	94.3 ± 1.7 a	231	93.1 ± 1.7 a
Egg	51	8.0 ± 0.0 a	66	7.8 ± 0.1 a	61	5.7 ± 0.1 b
Larva	47	2.3 ± 0.1 a	62	2.2 ± 0.1 a	61	2.7 ± 0.1 b
Protonymph	44	2.9 ± 0.1 a	59	2.0 ± 0.1 b	59	1.7 ± 0.1 c
Deutonymph	43	3.0 ± 0.1 a	57	3.3 ± 0.1 b	58	2.1 ± 0.1 c
Egg-to-adult	43	16.1 ± 0.1 a	57	15.3 ± 0.1 b	58	12.2 ± 0.1 c
Survival rate (%)	77	90.6 ± 3.3 a	77	87.0 ± 3.8 a	80	89.6 ± 3.4 a
Longevity	36	18.2 ± 1.6 a	51	17.3 ± 1.0 a	45	10.2 ± 1.0 b
Lifespan	41	31.5 ± 1.8 a	57	30.2 ± 1.3 a	51	21.1 ± 0.9 b
July						
Hatchability (%)	112	98.2 ± 1.3 a	106	99.1 ± 0.9 a	104	98.1 ± 1.3 a
Egg	65	4.7 ± 0.1 a	60	4.3 ± 0.1 b	58	3.3 ± 0.1 c
Larva	65	2.3 ± 0.1 a	60	2.1 ± 0.1 b	58	1.9 ± 0.1 c
Protonymph	65	2.0 ± 0.0 a	58	1.8 ± 0.1 b	57	1.4 ± 0.1 c
Deutonymph	64	1.9 ± 0.1 a	58	1.8 ± 0.1 a	54	1.8 ± 0.1 a
Egg-to-adult	64	11.0 ± 0.1 a	58	10.0 ± 0.1 b	54	8.5 ± 0.1 c
Survival rate (%)	75	96.9 ± 2.0 a	74	96.4 ± 2.2 a	76	92.9 ± 2.9 a
Longevity	61	13.9 ± 0.9 a	54	17.1 ± 0.8 b	50	16.1 ± 1.1 ab
Lifespan	62	24.7 ± 0.9 a	58	25.9 ± 1.0 a	54	23.2 ± 1.2 a
August						
Hatchability (%)	207	96.6 ± 1.3 a	146	93.8 ± 2.0 a	310	91.3 ± 1.6 a
Egg	68	4.0 ± 0.0 a	60	3.8 ± 0.1 a	62	3.4 ± 0.1 b
Larva	68	2.0 ± 0.0 a	56	1.4 ± 0.1 b	59	1.4 ± 0.1 b
Protonymph	67	1.2 ± 0.1 a	42	1.7 ± 0.1 b	56	1.2 ± 0.1 a
Deutonymph	65	2.1 ± 0.1 a	42	1.4 ± 0.1 b	52	1.2 ± 0.1 b
Egg-to-adult	65	9.2 ± 0.1 a	42	8.4 ± 0.1 b	52	7.2 ± 0.1 c
Survival rate (%)	78	92.9 ± 2.9 a	67	86.8 ± 4.1 a	78	79.6 ± 4.6 a
Longevity	48	15.5 ± 1.0 a	37	15.5 ± 1.0 a	50	8.4 ± 0.5 b
Lifespan	51	23.5 ± 1.1 a	42	21.7 ± 1.3 a	62	13.5 ± 0.7 b

Data are mean ± SEM, in days unless noted otherwise. Values with the same letter in rows are not significantly different (*P *> 0.05)

There were significant differences in the durations of the immature stages and in adult longevity and lifespan among different temperature scenarios during June (eggs: *F*_2, 175_ = 621.7; larvae: *F*_2, 167_ = 17.93; protonymphs: *F*_2, 159_ = 43.66; deutonymphs: *F*_2, 155_ = 64.03; egg-to-adult: *F*_2, 155_ = 497.4; longevity: *F*_2, 129_ = 14.95; lifespan: *F*_2, 149_ = 17.91; *P*_all_ < 0.0001) (Table 2). Post-hoc comparisons revealed no significant differences between the current and RCP2.6 scenarios in the lengths of the egg and larval periods and in adult longevity and lifespan (*P* > 0.05), but significant differences were found between these two scenarios for the duration of the protonymph (*P* < 0.0001) and deutonymph (*P* = 0.0055) stages and for total egg-to-adult period duration (*P *< 0.0001) (Table 2). The durations of the immature stages, as well as adult longevity and lifespan, of mites reared under the current scenario were all significantly different from those of mites reared under the RCP8.5 scenario (*P* < 0.0001). The immature development and adult longevity and lifespan were generally longer in mites reared under RCP2.6 than in those reared under RCP8.5 (protonymphs, *P* = 0.0343; others, *P* < 0.0001).

During July there were significant differences among different temperature scenarios in the durations of the egg incubation period (*F*_2, 180_ = 140.4, *P* < 0.0001), the larval period (*F*_2, 180_ = 13.26, *P *< 0.0001), the protonymph stage (*F*_2, 177_ = 34.5, *P* < 0.0001), and the egg-to-adult period (*F*_2, 173_ = 229.8, *P* < 0.0001), but not in the length of the deutonymph stage (*F*_2, 173_ = 1.466, *P* = 0.234). Adult longevity differed significantly among scenarios (*F*_2, 162_ = 3.316, *P* = 0.0388) but lifespan did not (*F*_2, 171_ = 1.656, *P* = 0.194). Post-hoc comparison showed that, with the exception of deutonymph period duration and lifespan (*P* > 0.05), all other developmental periods differed significantly among different temperature scenarios in July (*P* < 0.05).

In August there were significant differences among temperature scenarios in the durations of the immature stages and the adult longevity and lifespan of *T. evansi* (eggs: *F*_2, 187_ = 37.98; larvae: *F*_2, 180_ = 32.47; protonymphs: *F*_2, 162_ = 19.24; deutonymphs: *F*_2, 156_ = 32.06; egg-to-adult: *F*_2, 156_ = 265.6; longevity: *F*_2, 132_ = 27.36; lifespan: *F*_2, 152_ = 31.01; *P*_all_ < 0.0001). The larval, deutonymph, and egg-to-adult periods, but not the egg incubation period (*P* = 0.0571), were significantly longer in mites reared at the current temperature than in those reared at RCP2.6 (*P*_all_ < 0.0001), whereas the protonymph stage was significantly shorter in the former. The immature stages (except protonymphs) of mites reared under the current temperature were significantly longer than those of mites reared at RCP8.5. The egg incubation, protonymph, and egg-to-adult periods of mites reared at RCP8.5 were significantly shorter than those of mites reared at RCP2.6 (*P* < 0.0001). Adult longevity and lifespan did not differ significantly between the current and RCP2.6 scenarios (*P* > 0.05), but both were significantly greater under these two scenarios than under RCP8.5 (*P* < 0.0001). Male egg-to-adult duration was slightly shorter than that of females under all test conditions (data not shown).

### Reproductive phases, fecundity, and sex ratio

The durations of the reproductive phases (pre-oviposition period [PrOP], oviposition period [OP], and post-oviposition period [PsOP]), as well as fecundity (total eggs/female [TEF]), varied among different temperature scenarios (Table 3). In June there were significant differences among temperature scenarios in the durations of the reproductive phases and in fecundity (PrOP: *F*_2, 140_ = 7.148, *P* = 0.0011; OP: *F*_2, 129_ = 15.4, *P* < 0.0001; PsOP: *F*_2, 129_ = 5.931, *P* = 0.0034; TEF: *F*_2, 128_ = 13.31, *P *< 0.0001). Duration of the oviposition and post-oviposition periods, as well as total eggs/female, did not differ significantly between mites under the current and RCP2.6 scenarios during June (*P* > 0.05), whereas the pre-oviposition period was significantly longer under current conditions (*P* = 0.0182). During June, mites reared under RCP8.5 had a significantly shorter oviposition period and fewer total eggs/female than those under the current and RCP2.6 scenarios (*P *< 0.0001). There were no significant differences in sex ratio among different temperature scenarios in June (χ^2^, *P* > 0.05).

**Table 3:** Effects of simulated global warming scenarios on length of reproductive phases, fecundity, and sex ratio of *Tetranychus evansi*

Parameter	Global warming scenario					
	*n*	Current	*n*	RCP2.6	*n*	RCP8.5
June						
Pre-oviposition	36	1.2 ± 0.1 a	56	1.0 ± 0.0 b	51	1.3 ± 0.1 a
Oviposition	36	16.0 ± 1.5 a	51	15.0 ± 0.9 a	45	8.5 ± 0.8 b
Post-oviposition	36	1.0 ± 0.2 ab	51	1.3 ± 0.2 a	45	0.5 ± 0.2 b
Eggs/female (no.)	36	93.1 ± 8.5 a	51	90.6 ± 5.9 a	45	50.6 ± 5.3 b
♀ ratio (%)	197	87.3 ± 2.4 a	177	85.9 ± 2.6 a	171	87.7 ± 2.5 a
July						
Pre-oviposition	63	1.3 ± 0.1 a	56	0.9 ± 0.0 b	54	1.0 ± 0.1 b
Oviposition	61	12.0 ± 0.8 a	54	15.4 ± 0.7 b	50	14.2 ± 1.0 ab
Post-oviposition	61	0.6 ± 0.2 a	54	0.9 ± 0.2 a	50	0.9 ± 0.2 a
Eggs/female (no.)	61	78.2 ± 5.7 a	54	105.8 ± 5.6 b	50	108.8 ± 8.1 b
♀ ratio (%)	442	91.4 ± 1.3 a	433	91.7 ± 1.3 a	383	90.6 ± 1.5 a
August						
Pre-oviposition	65	1.1 ± 0.0 a	40	1.0 ± 0.0 b	50	1.0 ± 0.1 ab
Oviposition	48	13.5 ± 1.0 a	37	14.0 ± 0.9 a	50	7.0 ± 0.5 b
Post-oviposition	48	0.9 ± 0.2 a	37	0.6 ± 0.2 a	50	0.4 ± 0.1 a
Eggs/female (no.)	48	89.6 ± 4.9 a	37	97.1 ± 5.5 a	50	45.4 ± 3.6 b
♀ ratio (%)	425	83.5 ± 1.8 a	292	86.3 ± 2.0 a	331	79.1 ± 2.2 a

Data are mean ± SEM, in days unless otherwise stated. Values with the same letter in rows are not significantly different (*P* > 0.05)

In July, there were significant differences among temperature scenarios in the durations of the pre-oviposition and oviposition periods, as well as in total eggs/female, but not in the post-oviposition period (PrOP: *F*_2, 170_ = 12.85, *P* < 0.0001; OP: *F*_2, 162_ = 5.052, *P* = 0.0074; PsOP: *F*_2, 162_ = 1.101, *P* = 0.335; TEF: *F*_2, 162_ = 7.182, *P *= 0.0010). In July, mites under RCP2.6 or RCP8.5 had pre-oviposition periods significantly shorter than those under the current temperature; the oviposition period under RCP2.6 was significantly longer than that under the current scenario but similar to that under RCP8.5, and total eggs/female under RCP2.6 and RCP8.5 was significantly greater than under the current scenario. There were no significant differences in sex ratio among different temperature scenarios in July (χ^2^, *P* > 0.05).

In a pattern similar to that in July, there were significant differences among temperature scenarios in the durations of the pre-oviposition and oviposition periods and in total eggs/female, but not in the duration of the post-oviposition period (PrOP: *F*_2, 152_ = 3.785, *P* = 0.0249; OP: *F*_2, 132_ = 28.11, *P* < 0.0001; PsOP: *F*_2, 132_ = 1.89, *P *= 0.155; TEF: *F*_2, 132_ = 37.13, *P* < 0.0001). The oviposition period was significantly shorter, and total eggs/female significantly fewer, under RCP8.5 than under the other two temperature scenarios (*P* < 0.0001). The ratio of females to males under RCP8.5 was slightly smaller than those under the current and RCP2.6 scenarios, but the differences were not significant (χ^2^, *P* > 0.05).

### Life tables

**Fig. 3:**
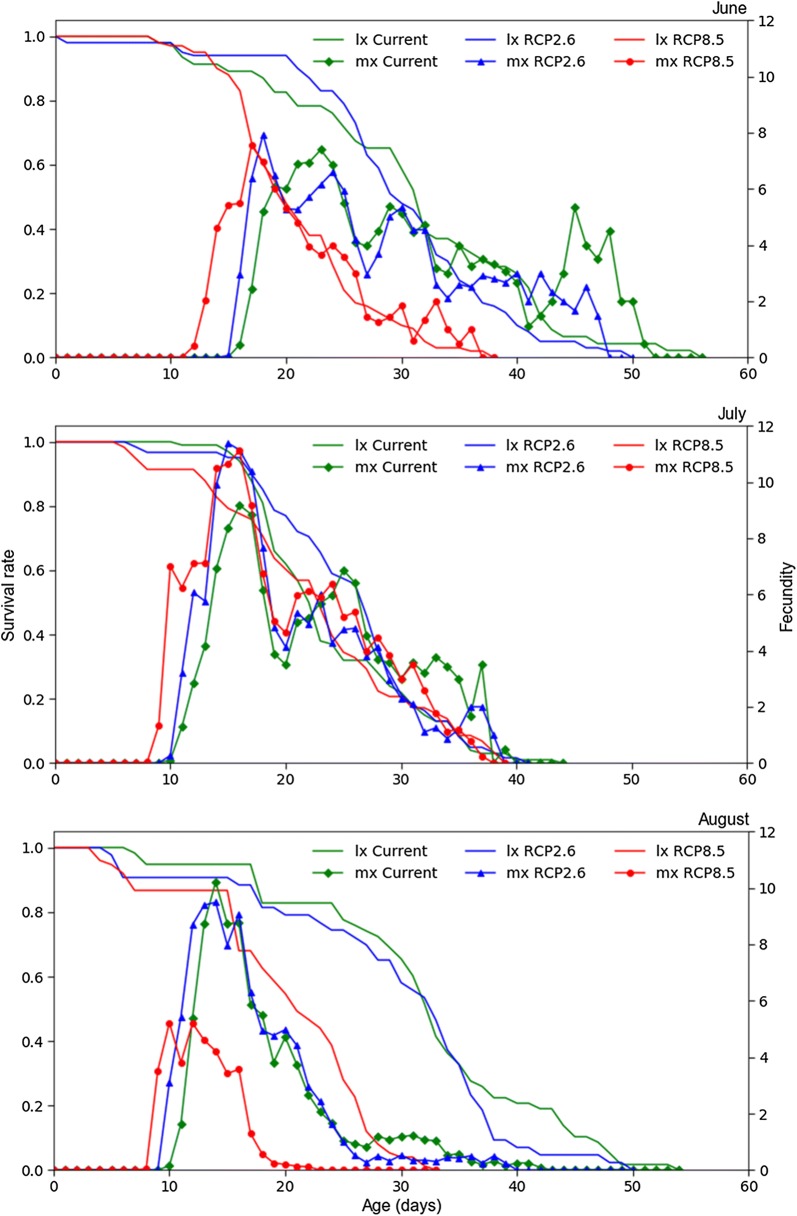
Age-specific survival rate (*l*_*x*_) and age-specific fecundity (*m*_*x*_) of *Tetranychus evansi* under different global warming scenarios

A steeper decline in the age-specific survival rate (*l*_*x*_) curve was observed in mites under RCP8.5 in June and August (Fig. 3) owing to higher mortalities among adults in both months. In June and August the declines under the current and RCP2.6 scenarios were similar. In July, the *l*_*x*_ curves all declined in a similar manner. The age-specific fecundity (*m*_*x*_, females/female/day) curves revealed that the onset of reproduction and the reproduction peak approached earlier in mites reared under RCP8.5 than in mites reared under RCP2.6 or the current scenario, regardless of the month. The highest peak in June occurred under RCP2.6 (7.8 females/female/day), in July under RCP2.6 (11.2 females/female/day), and in August under the current scenario (10 females/female/day).

In June, there were significant differences among temperature scenarios of current and RCP8.5 in all life table parameters (Table 4). The mean values of *R*_0_ (41.5), and *T* (17.8) were lower under RCP8.5 and differed significantly from those under RCP2.6. The intrinsic rate of increase and finite rate of increase under RCP8.5 were significantly higher than those under current scenario. To a similar extent, in July there were significant differences among temperature scenarios in the life table values; the means of the life-table values under RCP2.6 and RCP8.5 differed significantly from those under the current temperature. In August, there were significant differences among temperature scenarios of current and RCP2.6 in *T* (*P* < 0.0001), *r*_m_ (*P* = 0.0085), *λ* (*P* = 0.0085) and *D*_*t*_ (*P* = 0.0087) but not *R*_0_ (*P* > 0.05).

**Table 4:** Effects of simulated global warming scenarios on life-table parameters of *Tetranychus evansi*

Parameter	Global warming scenario					
	*n*	Current	*n*	RCP2.6	*n*	RCP8.5
June						
*R*_0_	41	72.2 ± 8.8 a	59	73.3 ± 6.5 a	55	39.3 ± 5.0 b
*T*		23.9 ± 0.3 a		22.2 ± 0.2 b		17.8 ± 0.3 c
*r*_m_		0.178 ± 0.004 a		0.193 ± 0.004 ab		0.205 ± 0.006 b
*λ*		1.195 ± 0.006 a		1.213 ± 0.004 ab		1.228 ± 0.007 b
*D*_*t*_		3.9 ± 0.1 a		3.6 ± 0.1 ab		3.4 ± 0.1 b
July						
*R*_0_	67	70.1 ± 5.8 a	59	93.6 ± 6.5 b	53	90.6 ± 8.8 ab
*T*		17.3 ± 0.3 a		16.7 ± 0.1 a		15.1 ± 0.2 b
*r*_m_		0.245 ± 0.005 a		0.272 ± 0.004 b		0.298 ± 0.006 c
*λ*		1.278 ± 0.006 a		1.312 ± 0.005 b		1.347 ± 0.008 c
*D*_*t*_		2.8 ± 0.1 a		2.6 ± 0.0 b		2.3 ± 0.0 c
August						
*R*_0_	58	73.9 ± 6.0 a	43	77.5 ± 7.5 a	75	30.1 ± 3.4 b
*T*		16.4 ± 0.2 a		15.3 ± 0.1 b		12.9 ± 0.1 c
*r*_m_		0.262 ± 0.004 a		0.283 ± 0.006 b		0.264 ± 0.008 ab
*λ*		1.300 ± 0.006 a		1.328 ± 0.008 b		1.302 ± 0.011 ab
*D*_*t*_		2.6 ± 0.0 a		2.4 ± 0.1 b		2.6 ± 0.1 ab

Data are mean ± SEM. Values with the same letter in rows are not significantly different at *P *< 0.05 by using paired bootstrap test

*R*_0_, net reproductive rate; *T*, generation time; *r*_m_, intrinsic rate of population increase; *λ*, finite rate of population increase; *D*_*t*_, population doubling time

## Discussion

The tomato red spider mite, *T. evansi*, is a serious agricultural pest with high invasion potential and is currently spread over many countries worldwide. Unlike other *Tetranychus* species, *T. evansi* develops at a range of temperature between 12 and 45 °C; it has a high thermal optimum (~ 35 to 43 °C) and a high rate of population increase (*r*_m_ = ~ 0.4 at 35 °C) [15, 22, 23]. These features enhance the ability of *T. evansi* to invade and adapt to a variety of geographical areas with varying climates and to become an emerging agricultural pest [12, 31]. It is therefore not surprising that we found here that *T. evansi* developed and reproduced even under the 2100 RCP8.5 scenario, in which the temperature was 3.7 °C above the current temperature and the maximum temperature experienced by the mites was 41.1 °C, during August. In general, development was favored by high temperatures (i.e., faster development with increased temperature), but reproduction was still high within moderate temperature ranges (i.e., under the current and RCP2.6 scenarios).

### Development and reproduction

The egg hatchability of *T. evansi* decreased slightly (but not significantly across scenarios within months) with increasing temperature and was as low as 91.3% at RCP8.5 in August. Different strains of *T. evansi* reared at a constant range of temperatures (15 to 35 °C) have shown 95% to 99% hatchability [23]. Our results suggested that the hatchability, as well as several life-history traits, of *T. evansi* exposed to natural fluctuations in temperature to high levels in August (peaking at more than 40 °C under RCP8.5) might be hindered. In a manner similar to other mite species, *T. evansi* generally developed faster as the temperature increased. Under the current temperature scenario, the egg-to-adult period was 16.1 days in June, 11.0 days in July, and 9.2 days in August, when the monthly average temperatures were 22.4, 25.4, and 27.1 °C, respectively. The egg-to-adult period was ~ 1 day shorter under RCP2.6 (+ 1 °C) than in the current scenario. Under RCP8.5 (+ 3.7 °C), the egg-to-adult period was 2 to 4 days shorter than in the current scenario and 1 to 3 days shorter than under RCP2.6. Under semi-field conditions in Mauritius, Moutia [13] reported that the egg-to-adult period of *T. evansi* was about 6.5 days in summer (mean temperature 22.8 °C) and 18.5 days in winter (mean temperature 19.4 °C). The value of 6.5 days [13] is much shorter than the values we obtained here—for example, under the current scenario in June, when the average temperature was ~ 22 °C. This difference undoubtedly is a result of differences in the diurnal temperature fluctuation patterns between the tropical climate of Mauritius (20.3484° S, 57.5522° E) and the temperate to humid subtropical climate of Tokyo (35.6895° N, 139.6917° E). Our *T. evansi* males developed slightly faster than the females (data not shown), and this trend was common among all conditions tested. This phenology is common among *Tetranychus* species and has been reported before in *T. evansi* [15, 23]. The immature survival rate (i.e., rate of survival to adulthood) ranged from 80% to 97%, with no significant effects of different temperature scenarios within the same month. To a similar extent, different strains of *T. evansi* reared at temperatures between 15 and 35 °C have survival rates between 88 and 98%, with no significant differences [23].

The lifespan of mites reared at RCP8.5 in June was about two-thirds those of mites reared under the other scenarios, and the total eggs/female was about half that under the current and RCP2.6 scenarios. This trend was reversed during July, in which month the lifespan was similar among the different temperature scenarios. Interestingly, total eggs/female during July was significantly greater for females at RCP2.6 (~ 106 eggs/female) and RCP8.5 (~ 109 eggs/female) than under the current scenario (~ 78 eggs/female). In accordance with our experimental design, whereby the mites were introduced to each climate change scenario at the beginning of June and their offspring were used in the experiment the following month, the mites under RCP2.6 and RCP8.5 may have had the advantage of being adapted to temperature increases. Nevertheless, with the further increase in temperature during August under RCP8.5 the mites suffered a huge reduction in their lifespans and reproduction as compared with under other scenarios (see Tables 2, 3, 4). This can be explained by the maximum thermal limit for *T. evansi*, which might have been reached under RCP8.5 in August, resulting in large mortality rates among adults (Fig. 3). The percentage of females among the offspring ranged from 79% to 92% (see Table 3). A female-biased sex ratio is common among different strains of *T. evansi*, and to some extent it is higher than in other *Tetranychus* species [14, 15, 22, 23].

### Life tables

Life tables are very convenient tools for assessing environmental effects on population development, survival, and reproduction [38] The net reproductive rate (females/female/generation) was highest under current and RCP2.6 in June and August, but it differed significantly from that in the current scenario in July and from that under RCP8.5 only in June and August (see Table 4). Generation time (days) was shortest under RCP8.5 and ranged from 17.8 days in June to 12.9 days in August. The intrinsic rate of population increase was, in general, higher at increased temperature owing to the short development time and early peak reproduction. This might also explain the wide temperature range at which *T. evansi* is able to develop and cause economic crop losses. The finite rate of increase (females/female/day) displayed a trend similar to *r*_m_. Population doubling times were similar between RCP2.6 and RCP8.5 temperature scenarios in June, significantly shorter under the two scenarios in July, and similar between current and RCP8.5 and between RCP2.6 and RCP8.5 in August. Because of the fluctuating nature of temperature regimens that mimic natural conditions, the life-history and life-table trait measurements that we obtained here are, to some extent, different from those in previous reports that have relied solely on laboratory studies at constant temperatures [15, 22, 23]. Environmental factors other than temperature might be involved (e.g., host plant, see Murungi et al. [39]). However, these differences among results further highlight the importance of and the need for gathering biological measurements under conditions that mimic the natural diurnal fluctuations in environmental factors such as temperature.

## Conclusion

Invasive alien pest species pose great challenges to world agriculture, biodiversity, and ecosystems. Empirical evidence of the likely impact of global warming on agricultural pests is essential. Our findings indicated that *T. evansi* is able to rapidly adapt to the increases in temperature; therefore, with long-term adaptation, the mite will be able to spread more widely and to a broad range of environmental temperatures. As well as possessing the advantages of a high thermal optimum, short generation time, and high rate of population growth, *T. evansi*—as is becoming evident in other organisms—might have already undergone what is known as “micro-evolutionary change in situ”; through its journey from its native range to almost everywhere around the world this species may already have encountered large environmental variations [1, 6, 7, 40, 41]. This speculation is supported by the fact that *T. evansi* has already spread to a geographical area that has climatic gradients different from those in its native habit [12]. Our findings and previous modeling results [31] are in agreement that the greatest *T. evansi* threat will be shifted to higher latitudes. However, it might be difficult to generalize our findings to other pest mites, because development and reproduction temperatures are species-specific and have to be assessed on an individual species basis.

## Materials and methods

### Mite culture

The founder population of *T. evansi* was originally collected from black nightshade plant (*Solanum nigrum* L.) in Tokyo (Japan, 35°35′ N, 139°36′ E) in November 2006 and was maintained on black nightshade at 25 ± 1 °C under a 16:8-h (light:dark) photoperiod at the Laboratory of Applied Entomology and Zoology, Faculty of Agriculture, Ibaraki University, Japan [17, 24]. A colony of *T. evansi* was brought to our laboratory at Tokyo University of Agriculture and Technology in May 2017 and thereafter maintained on detached leaves of eggplant (*Solanum melongena* L. cv. Senryo #2) at 25 ± 1 °C with a 16:8-h photoperiod. The mites were maintained on eggplant for about 3 months before they were used in the experiments.

### Climate data

Temperature data for Tokyo in 2016 (June, July, and August) in 10-min measurement intervals were obtained from the Japan Meteorological Agency (http://www.jma.go.jp/jma/index.html). To examine the effects of predicted global warming, the average temperature increase values in two scenarios defined by the IPCC [5] were used. Under RCP2.6 the temperature was projected to increase by 1 °C, and under RCP8.5 by 3.7 °C, on average. An environmental simulation system (ESS) was used to simulate temperature data and create the three scenarios. ESS is a computer-based closed system for simulating natural climate conditions [37, 42, 43]. Briefly, the system software created a schedule of 10-min intervals with corresponding set values of air temperature (set temperature, TSVs) and continuously measured the process values of air temperature (measured temperature, TPVs). TPV was adjusted to TSV by switching on or off an air heater (TSR210-A; Tescom, Tokyo, Japan) and a refrigerator (JF-NU40B; Haier Japan Sales, Osaka, Japan) every 10 min. The daily natural photoperiod was also created by the system by turning on or off light-emitting diodes. Relative humidity was not simulated but was recorded throughout the experiments (Additional file 1: Figure S1).

### Development and reproduction

Three units of the ESS were used simultaneously. Each unit was assigned to one of the temperature scenarios. A random-aged egg-laying adult females of *T. evansi* were introduced onto a detached eggplant leaf (ca. 40 × 40 mm) placed upside down on a water-saturated cotton pad in a polystyrene square Petri dish (100 × 15 mm). Then, on 1 June, each dish was transferred to one of the ESS units; the females were left to lay eggs for 24 h and then removed. The eggs were categorized into two groups: one group was kept on the same detached leaf until hatching and used to calculate hatchability percentages. The other group (approximately 80 eggs) was transferred separately onto eggplant leaf discs (1 cm in diameter) and observed daily throughout the mites’ whole lifespan. Leaf discs were replaced every 3 days or when necessary. Upon adult female emergence, one adult male was introduced for copulation. To determine the sex ratio, 2 or 3 days after the onset of oviposition, the eggs laid by females in each treatment during 2 consecutive days were collected and transferred to a new detached eggplant leaf. The collected eggs were kept to develop to adulthood and then sexed. These adults were maintained under the same conditions and used to produce eggs for the consecutive-month experiment. For example, the June progeny under the current scenario were transferred to a new detached leaf on 1 July under the same scenario and allowed to lay eggs for 24 h; the adults were then discarded and the eggs treated as previously. The same observations and experimental procedures were applied to other months and scenarios.

### Statistical analysis

Pearson’s correlation coefficient was used to examine the relationship between TSVs and TPVs as evidence of simulation accuracy. Percentages (hatchability, survival to adulthood, and sex ratio) were compared by using a Chi-squared test with Bonferroni correction. Two-way analysis of variance (ANOVA) was used to test the effects of month and temperature scenario on development time (egg-to-adult), lifespan, and reproduction (eggs/female). One-way ANOVA with Tukey’s post hoc test was used for means separation with α = 0.05. The development and reproduction data were square-root transformed to meet assumptions of normality when necessary and retransformed for representation purposes. The data for development, survival rate, longevity and female daily fecundity were analyzed according to the age-stage, two-sex life table approach using the computer program TWOSEX-MSChart [44–46]. The following parameters were calculated as described by Chi & Liu [44]: age-stage-specific survival rate (*s*_*xj*_, the probability that a newly laid egg will survive to age *x* and stage *j*), age-specific survival rate (*l*_*x*_, the percentage of females alive at age *x*), age-specific fecundity (*m*_*x*_, the number of female offspring produced by a female in a unit of time), age-stage specific fecundity (*f*_*xj*_, the mean fecundity of females at age *x*), intrinsic rate of natural increase (*r*), finite rate of increase (*λ*, the number of times the population multiplies in a unit of time), net reproductive rate (*R*_0_, number of female offspring/female/generation), mean generation time (*T*, mean age of mothers at time of birth of female offspring) and population doubling time (*D*_*t*_, the time needed for the population to double). The age-specific survival rate (*l*_*x*_) and age-specific fecundity (*m*_*x*_) are calculated as:

$$l_{x} = \mathop \sum \limits_{j = 1}^{k} s_{xj}$$

$$m_{x} = \frac{{\mathop \sum \nolimits_{j = 1}^{k} s_{xj} f_{xj} }}{{\mathop \sum \nolimits_{j = 1}^{k} s_{xj} }}$$

where *k* is the number of life stages. The reproductive rate (*R*_0_) is calculated as follows:

$$R_{0} = \mathop \sum \limits_{x = 0}^{\infty } l_{x} m_{x}$$

The intrinsic rate of population increase (*r*_m_) was calculated by using the Euler–Lotka equation as follows:

$$\mathop \sum \limits_{x = 0}^{\infty } e^{{ - r_{m} \left( {x + 1} \right)}} l_{x} m_{x} = 1$$

where *x* is a female age in days. The finite rate of increase (*λ*), mean generation time (*T*) and population doubling time (*D*_*t*_) are calculated as follows:

$$T = \ln \frac{{R_{0} }}{{r_{m} }},$$

$$\lambda = e^{{r_{m} }} ,$$

$$D_{t} = \frac{\ln 2}{{r_{m} }}.$$

The bootstrap procedure (B = 100,000) was used to estimate means and standard errors for the life tables parameters and the paired bootstrap test was used to compare different treatment based on the confidence interval of the differences using TWOSEX-MSChart [47–49]. ANOVAs were performed in R v. 3.4.0 software [50]. Pearson’s correlation coefficient was determined by using scipy.stats.pearsonr in Python [51]. Data were visualized with Matplotlib in Python [52].

## Supplementary information

**Additional file 1: Figure S1.** Relative humidity (%) measurements made during the experiments.

## Data Availability

The data used and/or analyzed during the current study are available from the corresponding authors on reasonable request.

## Abbreviations

ESS: environmental simulation system
fig: figure
IPCC: intergovernmental panel on climate change
*l*_*x*_: age-specific survival rate
*m*_*x*_: age-specific fecundity
OP: oviposition period
PrOP: pre-oviposition period
PsOP: post-oviposition period
*R*_0_: net reproductive rate
*T*: generation time
*r*_m_: intrinsic rate of population increase
RCP: representative concentration pathway
TEF: total eggs/female
TPV: measured temperature
TSV: set temperature
*λ*: finite rate of population increase
*D*_*t*_: population doubling time
